# Supplementary material for: Heregulin Drives Endocrine Resistance by Altering IL-8 Expression in ER-Positive Breast Cancer
Source: Int J Mol Sci. 2020 Oct 19;21(20):7737. doi: 10.3390/ijms21207737 (PMC7589856; doi:10.3390/ijms21207737)
Supplement: Supplementary file 1 [file ijms-21-07737-s001.pdf]

Brief Report

Heregulin overexpression drives endocrine resistance by altering IL-8 expression in ER-positive breast cancer

SUPPLEMENTARY INFORMATION

C+	C+	C+	C+	Blank	Acrp30	AgRP	Angiopoietin-2	Amphiregulin	Axl	bFGF	b-NGF	BTC	CCL-28
C-	C-	C-	C-	Blank	Acrp30	AgRP	Angiopoietin-2	Amphiregulin	Axl	bFGF	b-NGF	BTC	CCL-28
CTACK	Dtk	EGFR	ENA-78	Fas/TNFR SF6	FGF-4	FGF-9	GCSF	GITR-Ligand	GITR	GRO	GRO-α	HCC-4	HGF
CTACK	Dtk	EGFR	ENA-78	Fas/TNFR SF6	FGF-4	FGF-9	GCSF	GITR-Ligand	GITR	GRO	GRO-α	HCC-4	HGF
ICAM-1	ICAM-3	IGFBP-3	IGFBP-6	IGF-I SR	IL-1 R4/ST2	IL-1 RI	IL-11	IL-12 p40	IL-12 p70	IL-17	IL-2Rα	IL-6 R	IL-8
ICAM-1	ICAM-3	IGFBP-3	IGFBP-6	IGF-I SR	IL-1 R4/ST2	IL-1 RI	IL-11	IL-12 p40	IL-12 p70	IL-17	IL-2Rα	IL-6 R	IL-8
I-TAC	Lymphotactin	MIF	MIP-1α	MIP-1β	MIP-3β	MSPα	NT-4	Osteoprotegerin	Oncostatin M	PIGF	sgp130	sTNF RII	sTNF RI
I-TAC	Lymphotactin	MIF	MIP-1α	MIP-1β	MIP-3β	MSPα	NT-4	Osteoprotegerin	Oncostatin M	PIGF	sgp130	sTNF RII	sTNF RI
TECK	TIMP-1	TIMP-2	Thrombopoietin	TRAIL R3	TRAIL R4	uPAR	VEGF	VEGF-D	Blank	Blank	Blank	Blank	Blank
TECK	TIMP-1	TIMP-2	Thrombopoietin	TRAIL R3	TRAIL R4	uPAR	VEGF	VEGF-D	Blank	Blank	Blank	C+	C+

Figure S1. Cytokines map of the RayBio® Human Cytokine Antibody Array VII & 7.1
